# Coriander Honey Accelerates Human Osteoblast Differentiation and Matrix Mineralization via Intracellular Ca^2+^ Signaling

**DOI:** 10.3390/ph19070979

**Published:** 2026-06-24

**Authors:** Gregorio Bonsignore, Elia Ranzato, Simona Martinotti

**Affiliations:** DiSIT—Dipartimento di Scienze e Innovazione Tecnologica, University of Piemonte Orientale, Viale Teresa Michel 11, 15121 Alessandria, Italy; gregorio.bonsignore@uniupo.it (G.B.); elia.ranzato@uniupo.it (E.R.)

**Keywords:** coriander honey, human osteoblasts, intracellular calcium, osteogenesis

## Abstract

**Background/Objectives**: Managing bone diseases demands novel, natural compounds to bypass the heavy side effects of current therapies. Honey is well-known for its therapeutic traits, yet we know very little about how specific floral varieties impact bone tissue. This study confronts this gap by comparing how acacia, chestnut, and coriander honeys drive human osteoblast behavior in vitro. **Methods**: After mapping the phenolic/flavonoid profiles and antioxidant capacities of these honeys, we tested them on hFOB 1.19 human osteoblasts. We tracked cell migration via scratch assays and validated osteogenic maturation through Alkaline Phosphatase (ALP) activity and Alizarin Red (AR) mineralization over 7 days. Confocal time-lapse imaging with pharmacological inhibitors monitored intracellular calcium dynamics, while gene shifts were analyzed via qRT-PCR. **Results**: Coriander honey (CH) packed the highest polyphenol levels and antioxidant power. Biologically, while all honeys accelerated scratch closure, CH drove cell motility most potently. Remarkably, a 7-day treatment with these honeys sparked a significant and robust increase in ALP activity and mineralization, surpassing the osteogenic induction observed with standard osteoinductive media. Mechanistically, CH triggered a sharp [Ca^2+^] spike, relying on external calcium entry and IP3-dependent internal release via PLC activation. qRT-PCR confirmed this anabolic shift via OPG and OPN upregulation. **Conclusions**: Honey exerts pronounced multi-level osteopromotive effects at both the functional and transcriptional levels, tightly linked to its botanical source. Among the variants, coriander honey stands out for its exceptional ability to fast-track osteoblast migration, differentiation, and early mineral deposition. Therefore coriander honey represents a promising in vitro candidate that warrants further preclinical evaluation for bone repair applications.

## 1. Introduction

Bone is a highly specialized and composite tissue continuously and finely remodeled during life. Such a complex biological process relies on the subtle balance between specific cells devoted to the formation of new bone—the osteoblasts—and those responsible for resorbing the aged or damaged tissue, the osteoclasts [1]. The impairment of this delicate balance gives rise to a series of bone pathologies and most notably to osteoporosis, characterized by the progressive decrease in mass and, consequently, increased fragility fractures. Currently, bone regenerative strategies and treatments for these debilitating diseases have serious drawbacks, regarding side effects and high cost and/or, in some cases, limited long-term efficacy [2]. It was this challenge that gave rise to growing interest in the testing of alternative, naturally derived compounds with osteoinductive and osteoprotective potential [3].

Honey is a complex natural matrix rich in bioactive compounds, produced and hived by honeybees, used for thousands of years in many traditional medicines around the world for its several beneficial properties [4]. Among its well-established properties are its potent antimicrobial, anti-inflammatory, and remarkable wound-healing capacities [5]. Although most scientific studies conducted so far have been heavily concentrated on Manuka honey, mainly for its strong non-peroxide antimicrobial activity mediated by methylglyoxal, this has often obscured the therapeutic potential of other varieties.

It is important, above all, to underline the fact that honey is not one homogeneous product. The composition and properties are directly related to the floral source, geographical origin, and season of gathering of honey. Honeys originating from different nectars have multiple classes of bioactive compounds, including a wide array of flavonoids, phenolic acids, enzymes, and vitamins, responsible for their specific antioxidant, anti-inflammatory, and biological activities [6]. Such deep chemical diversity relates directly to the fact that the beneficial effects demonstrated by one type of honey could well not be emulated by another, reinforcing once again the need for specific and focused investigations.

Although honey has been consumed traditionally for centuries and its biochemical richness from a variety of floral sources has been well-established, the floral varieties’ effects on bone cells are relatively less studied, particularly for honeys other than Manuka [7].

For honeys other than Manuka, their ability to impact proliferation, differentiation, and subsequent matrix mineralization by osteoblasts has not been systematically characterized. This represents an important knowledge gap in identifying new natural agents with distinct osteopromotive properties. While the general antioxidant and anti-inflammatory properties of honey have been extensively documented, a critical gap remains in our understanding of how specific monofloral varieties differentially modulate human osteoblast maturation. In particular, the precise intracellular signaling cascades—such as calcium signaling dynamics—and the targeted gene expression profiles triggered by unique honey phytocomplexes during early bone differentiation remain virtually unexplored.

Therefore, this work aims to establish a rigorous comparative screening of acacia, chestnut, and coriander honeys on human osteoblast activation. Moving beyond generic bioactivity, we characterize the exact functional transitions and gene expression shifts driven by these honeys, identifying the precise PLC-IP3/Ca^2+^ signaling cascade as the core driver of the osteopromotive effect. Ultimately, this comprehensive evaluation delivers a novel pharmacological rationale to support the integration of selected monofloral honeys into biocompatible scaffolds for bone repair and hard tissue regeneration.

## 2. Results

### 2.1. Chemical Characterization of Honey

**Figure 1:**
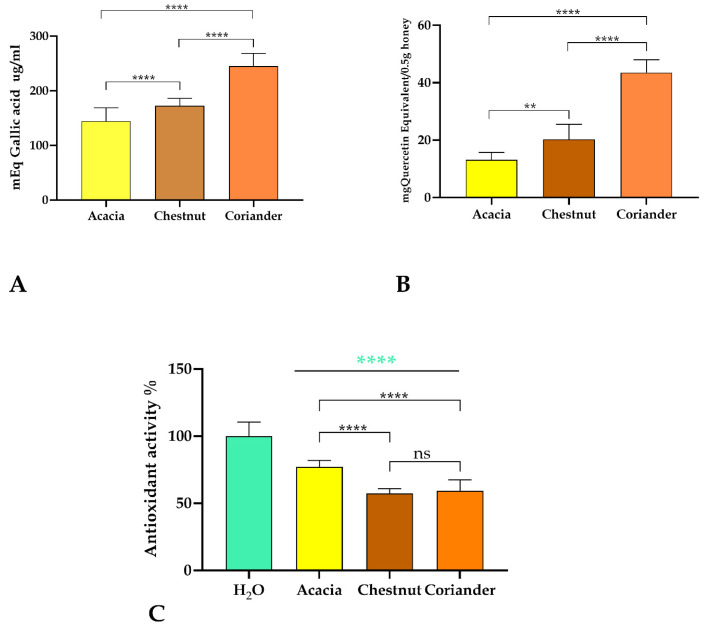
(**A**) Total phenolic content obtained with the Folin–Ciocalteu assay, expressed as mEq gallic Acid µg/mL. Bars represent mean ± SD of two independent experiments, each with n = 8. The mean of the control was set to 100. The statistical difference was determined by a one-way ANOVA followed by Tukey’s Multiple Comparison Test (**** *p* < 0.0001). (**B**) Total flavonoids content (TFC) obtained with Aluminum chloride assay, expressed as mEq quercetin µg/0.5 g honey (** *p* < 0.01). Statistics as in (**A**). (**C**) Free Radical Scavenging Activity obtained with 2,2-Diphenyl-1-picrylhydrazyl (DPPH) assay. Bars represent mean ± SD of two independent experiments, each with n = 8. The mean of water (H_2_O) was set to 100. The statistical difference between honey types and H_2_O was determined by a one-way ANOVA followed by Dunnett’s Multiple Comparison Test (**** *p* < 0.0001), statistical differences between honey types as in (**A**,**B**) (ns = not significant, **** *p* < 0.0001). The asterisk colors match the histogram bars and represent the statistical significance compared to the water control.

Acacia, chestnut, and coriander honeys were evaluated by determining their polyphenol and flavonoid content, as well as their antioxidant activity. Coriander honey showed the highest concentration of polyphenols and flavonoids, while acacia honey showed the lowest (Figure 1A,B). These data are in agreement with the recorded antioxidant activity, which was lowest for acacia honey and reached its peak in coriander honey (Figure 1C).

### 2.2. Scratch Wound Assay

The objective of this study was to evaluate the potential of acacia, chestnut and coriander honey to influence cellular processes critical for bone repair and regeneration.

**Figure 2:**
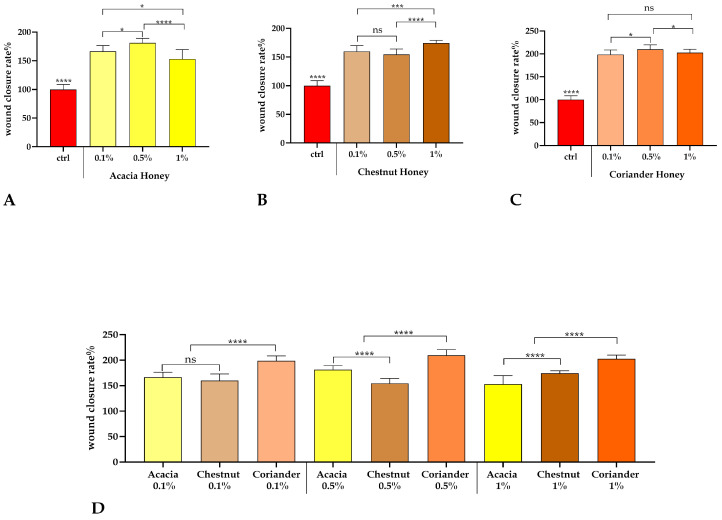
(**A**–**C**) Wound closure rate % in hFOB cells in control conditions (red bar) or exposed for 24 h to acacia honey, chestnut honey or coriander honey respectively. Bars represent mean ± SD (n = 15 from three independent experiments). Statistics indicate differences with respect to the control condition (**** *p* < 0.0001, One-way ANOVA followed by Dunnet’s multiple comparison test) or between different honey concentrations utilized (ns *p* > 0.05, * *p* < 0.05, *** *p* < 0.001, **** *p* < 0.0001, One-way ANOVA followed by Tukey’s test). (**D**) Comparison between honey types. Bars represent mean ± SD (n = 15 from three independent experiments). Statistics indicate differences between honey types at the same concentration (ns *p* > 0.05, **** *p* < 0.0001, One-way ANOVA followed by Tukey’s test).

To test this hypothesis, the effect of the considered honeys on the migratory capacity of human osteoblast cells (hOBs) was assessed using a standardized scratch wound healing assay. Cells were exposed to varying concentrations of honey types (0.1–0.5–1% *w*/*v*) and compared to untreated controls. Quantification of the remaining wound area at defined time points demonstrated that acacia, chestnut and coriander honey significantly promoted the rate of wound closure (Figure 2A–C).

Across the entire range of concentrations tested, coriander honey consistently outperformed the other two varieties. Comparative analysis at equivalent doses showed that coriander honey maintained the most robust effect (Figure 2D).

### 2.3. Functional Assessment of Osteogenic Potential: ALP Activity and Mineralization

To functionally validate the intrinsic osteopromotive capacity of honeys and assess their effect on osteoblast maturation, we performed the Alkaline Phosphatase (ALP) activity assay and the Alizarin Red (AR) staining assay.

ALP serves as a crucial early marker of osteoblast differentiation and matrix maturation. The quantification of ALP activity after 7 days of treatment demonstrated a significant induction of early-stage osteoblast differentiation across all honey samples, suggesting a robust pro-osteogenic effect.

**Figure 3:**
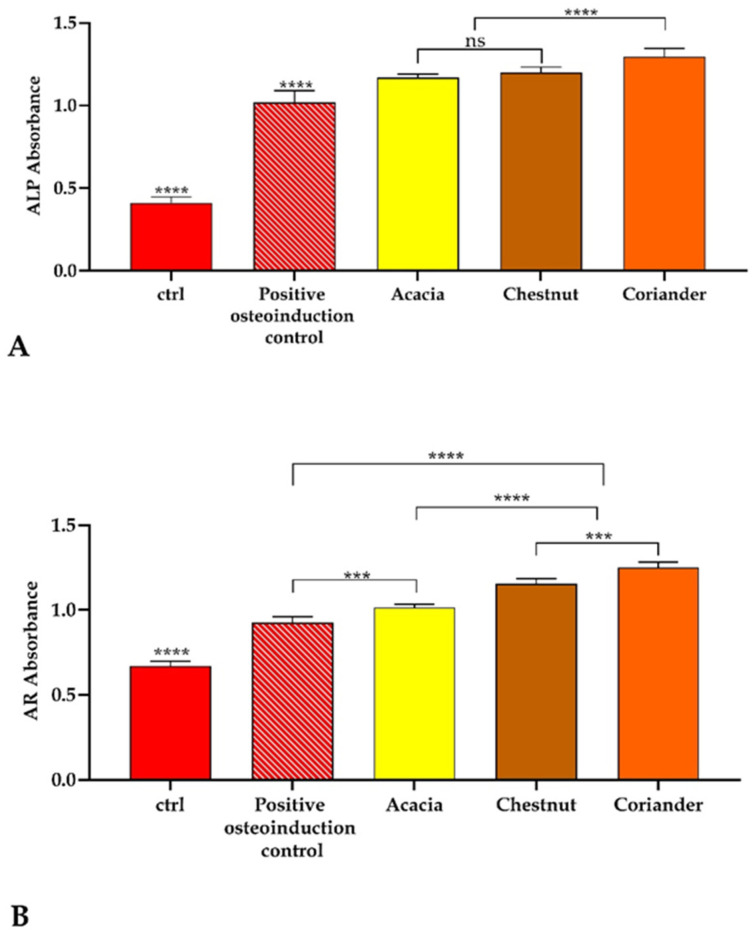
(**A**) Osteopromotive activity (ALP assays) carried out after 7 days of cell incubation. Cells were exposed to control conditions, osteoinductive medium, or 0.5% acacia, chestnut or coriander honey. Bars represent mean ± SD (n = 15 from three independent experiments). Statistics indicate differences with respect to the control condition (**** *p* < 0.0001, One-way ANOVA followed by Dunnet’s multiple comparison test), with respect to the positive osteoinductive control condition (**** *p* < 0.0001, One-way ANOVA followed by Dunnet’s multiple comparison test) or between different honey types (ns *p* > 0.05, **** *p* < 0.0001, One-way ANOVA followed by Tukey’s test). (**B**) Mineralization activities (AR assay) carried out after 7 days of cell incubation. Cells were exposed to control conditions, osteoinductive medium, or 0.5% acacia, chestnut or coriander honey. Bars represent mean ± SD (n = 15 from three independent experiments). Statistics indicate differences with respect to the control condition (**** *p* < 0.0001, One-way ANOVA followed by Dunnet’s multiple comparison test), with respect to the positive osteoinductive control condition (*** *p* < 0.001, **** *p* < 0.0001, One-way ANOVA followed by Dunnet’s multiple comparison test) or between different honey types (*** *p* < 0.001, **** *p* < 0.0001, One-way ANOVA followed by Tukey’s test).

Crucially, the efficacy of the honeys was directly compared to the standard osteoinductive (OI) medium (containing β-glycerophosphate and ascorbic acid, [8]) after 7 days of culture. In this head-to-head comparison, the ALP activity induced by each honey was significantly higher than that achieved by the full OI medium. This result demonstrates that honeys possess an intrinsic and powerful osteopromotive capacity that surpasses the established chemical induction regimen in the early stage of differentiation (Figure 3A).

The ability of osteoblasts to deposit a calcified matrix was assessed via Alizarin Red staining (AR), a marker of late-stage differentiation/early mineralization.

The observation of increased AR staining after 7 days is particularly significant, as mineralization is typically considered a late-stage event occurring after several weeks in culture.

The comparison with the standard OI medium was also performed for the mineralization assay. Quantification showed that all tested honeys induced AR staining levels that were significantly higher than those achieved by the standard OI medium alone (Figure 3B).

Notably, the effect of CH was the most potent, showing the highest level of calcified nodules among all treatments. This rapid and pronounced increase in mineralization at this early time point underscores the potent, expediting effect of coriander honey on the entire osteogenic differentiation process. Specifically, the data indicates that coriander honey positively influences the osteogenic pathway by accelerating the transition to matrix deposition more effectively than the standard chemical cocktail.

Collectively, the dual increase in both the early marker (ALP) and the mineralization marker (AR) at a short time point, showing superior performance compared to the established OI chemical medium, strongly supports the hypothesis that coriander honey possesses the potential to not only initiate but also to expedite the key phases of bone formation, behaving favorably even when benchmarked against established osteoinductive chemical cocktails.

Given its unique chemical profile and demonstrated biological activities, coriander honey was prioritized for further investigation to elucidate the underlying molecular and cellular pathways governing these observed biological responses.

### 2.4. Coriander Honey Modulates Intracellular Calcium (Ca^2+^) Dynamics in hFOBs

Intracellular calcium (Ca^2+^) is a universal second messenger, critically regulating many cellular processes in osteoblasts such as proliferation, differentiation, matrix mineralization and the expression of genes that regulate bone homeostasis.

**Figure 4:**
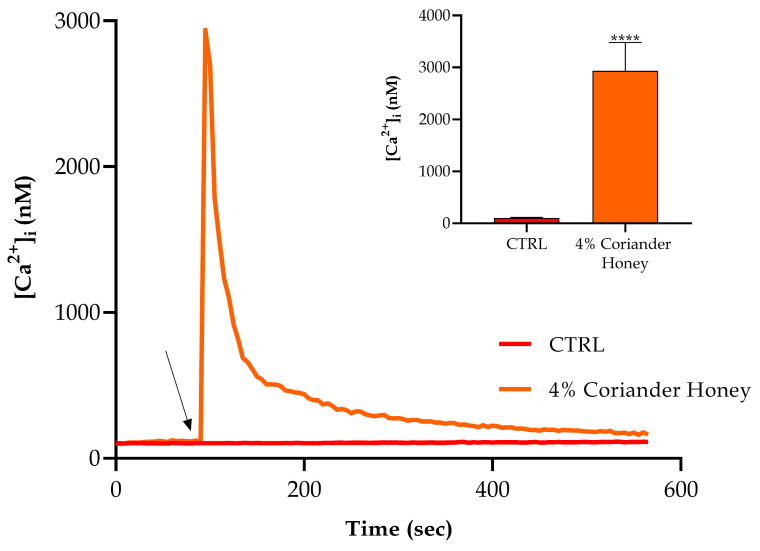
Variation of [Ca^2+^]_i_ after coriander honey-treatment recorded at 5 s intervals in the hFOB cell line. The arrow shows coriander honey addition after 60 s. Data are means of [Ca^2+^]_i_ traces recorded in different cells. Number of cells: 40 cells from three experiments for each cell line. Insert. Means ± SEM of Ca^2+^ peak response. Number of cells as before. Statistics indicate differences between control and coriander honey condition (**** *p* < 0.0001, Unpaired *t*-Test with Welch’s correction).

The proper control of Ca^2+^ oscillations is therefore fundamental to the maintenance of osteoblast function and, by consequence, of bone health. Our studies on the effects of coriander honey in human fetal osteoblast cells (hFOBs) evidenced an effect on their intracellular Ca^2+^ signaling. 4% (*v*/*v*) coriander honey consistently evoked a significant and rapid increase in the intracellular Ca^2+^ concentration ([Ca^2+^]_i_) in hFOBs (Figure 4).

Such increase presented as a rapid rise to a well-defined peak level, followed by a relatively rapid recovery towards baseline within 8 min. This transient nature of the Ca^2+^ signal argues for a regulated, specific signaling cell response rather than non-specific membrane damage or fatigue.

To identify the origin of the Ca^2+^ transient we performed the challenge with coriander honey under different extracellular conditions and in the presence of specific inhibitors of Ca^2+^ signaling pathways. When coriander honey was applied in a Ca^2+^-free extracellular solution the Ca^2+^ peak was dramatically reduced but not abolished.

**Figure 5:**
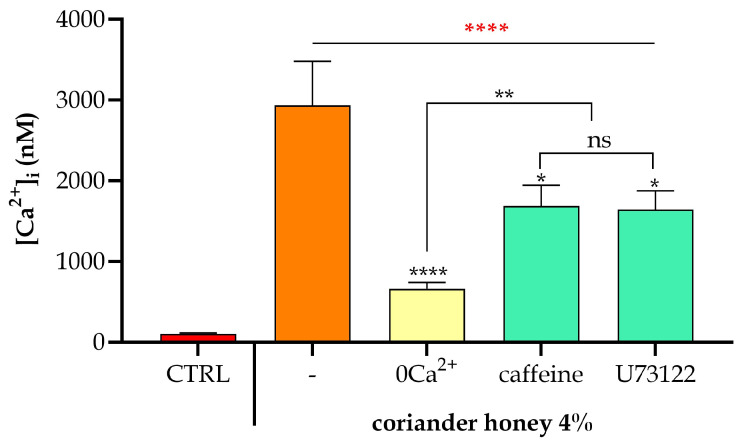
Variation of [Ca^2+^]_i_ after coriander honey-treatment, in the presence of different conditions, recorded at 5 s intervals in the hFOB cell line. Data are means ± SEM of Ca^2+^ peak response (n = 40 from three independent experiments). Statistics indicate differences with respect to the control condition (**** *p* < 0.0001, One-way ANOVA followed by Dunnet’s multiple comparison test), with respect to coriander honey treatment alone (* *p* < 0.05, **** *p* < 0.0001, One-way ANOVA followed by Dunnet’s multiple comparison test) or between coriander honey treatment in 0Ca^2+^ conditions (0Ca^2+^ = absence of extracellular calcium) or in presence of PLC-IP3 pathway inhibitors (ns *p* > 0.05, ** *p* < 0.01, One-way ANOVA followed by Tukey’s test). The color of the asterisks indicates the statistical significance relative to the condition oh honey without other drugs, matching the corresponding color of the histogram bar. The minus sign (“-”) denotes the coriander honey conditions without other drugs.

This observation points out that the size of the coriander honey-induced signal is mainly dependent on the entry of external Ca^2+^ (Figure 5). However, the residual, although smaller, Ca^2+^ peak observed in the absence of external Ca^2+^ also verifies a requisite contribution from intracellular Ca^2+^ stores, i.e., the endoplasmic reticulum (ER). To identify the molecular mechanism responsible for internal Ca^2+^ release we determined the effect of inhibitors of the main Ca^2+^ pathways (Figure 5).

A major role in the inositol trisphosphate (IP3) signaling pathway was demonstrated with U73122, a strong inhibitor of Phospholipase C (PLC). U73122 treatment significantly, though slightly, reduced the size of the coriander honey-induced Ca^2+^ peak (*p* < 0.001).

This result rigorously confirms that ER-store Ca^2+^ release is mediated by PLC-activated IP3 production; likewise, the involvement of the inositol trisphosphate receptor (IP3R) was confirmed by the fact that caffeine treatment induced a similar reduction in the Ca^2+^ peak.

Altogether, these pharmacological tools demonstrate that the coriander honey-induced transient Ca^2+^ signal in hFOBs is a highly regulated event, which depends critically on the activation of the PLC-IP3 pathway, which drives the rapid mobilization of internal Ca^2+^ stores, coupled to external Ca^2+^ influx to achieve the full size of the response. This dual mechanism suggests that an ingredient of the coriander honey is working via specific receptors at the cell surface that couple with this fundamental osteogenic signaling cascade.

### 2.5. Differential Gene Expression Analysis and Correlation with Phenotypic Outcomes

**Figure 6:**
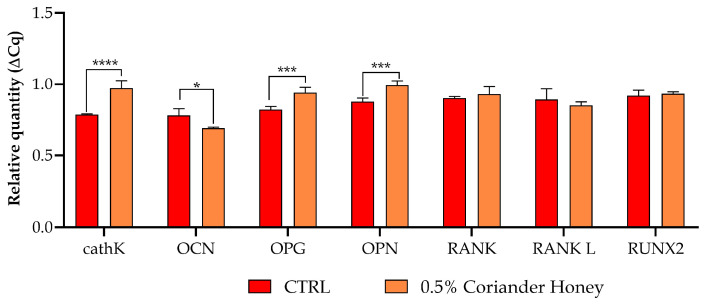
mRNA quantity determined by qRT-PCR of bone remodeling involved genes transcripts (cathepsin K (cathK), osteocalcin (OCN), osteoprotegerin (OPG), osteopontin (OPN), receptor activator of nuclear factor kappa β (RANK/RANKL) pathway, Runt-related transcription factor 2 (Runx2)) expressed as mean of relative expression ± SD (n = 3). Statistics indicate, for each mRNA group, differences between control (CTRL) and 0.5% coriander honey treatment (* *p* < 0.05, *** *p* < 0.001, **** *p* < 0.0001, Two-way ANOVA follow by Sidak’s test).

To gain further insights into the stimulatory effects observed in both osteoblast migration and Alkaline Phosphatase (ALP) modulation, we examined the transcriptional profiles of important genes involved in bone remodeling and osteogenic differentiation in hFOB 1.19 cells after treatment with coriander honey (Figure 6). Quantitative real-time PCR (qRT-PCR) analysis demonstrated a clear differential modulation of bone remodeling transcripts. Importantly, coriander honey induced a strong upregulation of pro-osteogenic and matrix-associated markers, particularly osteoprotegerin (OPG) and osteopontin (OPN). OPG is a major soluble decoy receptor that inhibits osteoclastogenesis and OPN is an important non-collagenous glycoprotein inextricably involved in cell attachment, substrate interaction and early matrix mineralization cascades.

Interestingly, we also observed a significant induction of cathepsin K (cathK). While cathepsin K is classically recognized as a major enzymatic driver of bone resorption, its localized expression by osteoblast lineages during active remodeling phases is essential for initial extracellular matrix processing and structural turnover. Crucially, the expression of upstream regulatory components in the receptor activator of nuclear factor kappa β (RANK/RANKL) cascade remained unaltered. Because the RANKL signaling axis is the primary driver of osteoclast recruitment, its absolute stability—combined with the significant upregulation of the soluble decoy receptor osteoprotegerin (OPG)—strongly indicates that CH shifts the local RANKL/OPG balance toward bone protection, thereby limiting the activation of osteoclastogenic or bone-resorbing pathways.

Regarding differentiation-specific markers, osteocalcin (OCN), a signature transcript of late-stage mature osteoblasts, exhibited a slight downregulation, while Runt-related transcription factor 2 (Runx2), the master transcription factor driving early osteoblastogenesis, showed no significant variations at this specific time point.

This precise gene expression profile strongly implies that, during early exposure windows, coriander honey does not forcefully accelerate terminal, post-mitotic differentiation; rather, it allows the cells to maintain a highly motile, pro-migratory progenitor phenotype. This transcriptional architecture perfectly mirrors and supports our functional outcomes and biochemical markers. The parallel induction of OPG and OPN—both deeply involved in matrix remodeling and cellular anchoring—provides a solid molecular rationale for the enhanced cell motility and accelerated gap closure documented in the scratch wound healing assays. These data suggest that coriander honey optimizes the capacity of human osteoblasts to interact with the underlying substrate, potentially through the synthesis of a specialized, OPN-enriched transitional matrix. Concurrently, the activation of ALP enzymatic activity combined with heightened OPG expression underscores the establishment of a robust pro-anabolic phenotype.

Taken together, while terminal calcification markers like OCN remain temporarily quiescent, the significant upregulation of OPG and OPN demonstrates that coriander honey successfully drives human osteoblasts into an active, early-to-mid osteoprogenitor state specialized in migration, protection against resorption, and structural matrix organization.

## 3. Discussion

Bypassing the heavy side effects and steep costs of current anti-osteoporotic therapies is a major driver behind the current push for natural, biocompatible alternatives [9]. While honey has been a staple of traditional medicine for centuries, mostly valued for its antimicrobial and wound-healing traits, its exact molecular impact on bone-forming cells has remained a bit of a black box [10].

This study directly addresses this gap through a comparative screening of three Italian monofloral honeys (acacia, chestnut, and coriander). What our data demonstrate is a multi-layered osteopromotive capacity that changes radically depending on the honey’s botanical origin. Among the tested varieties, coriander honey (CH) quickly stood out as the clear frontrunner, showing a superior ability to accelerate human osteoblast migration, early differentiation, and matrix calcification.

This biological performance tracks closely with the distinct phytochemical footprint of CH. Our chemical profiling showed that CH packed the highest concentrations of total phenolics and flavonoids [11], leading to the strongest antioxidant capacity of the group. In the context of bone remodeling, this antioxidant abundance matters immensely [12].

Chronic oxidative stress and the overproduction of reactive oxygen species (ROS) are notorious triggers for osteoblast apoptosis and flawed matrix cross-linking, both of which fuel bone fragility diseases like osteoporosis [13]. By delivering a dense pool of bioactive polyphenols, such as quercetin derivatives, CH likely builds a protective microenvironment. This shield optimizes osteoblast survival and preserves their functional machinery under stress.

This protective effect matches what we observed in the functional assays. Before any bone matrix can be built, osteoblasts must physically travel to the injury site to initiate repair [14]. While all three honeys accelerated gap closure, CH drove human osteoblast motility with exceptional punch. At the molecular level, this pro-migratory behavior makes sense when looking at our qRT-PCR data, which documented a significant upregulation of osteopontin (OPN). Beyond its classic role in mineralization, OPN functions as a non-collagenous structural glycoprotein that handles cell adhesion and anchoring [15]. The parallel rise in OPN expression and accelerated gap closure suggests that CH primes human osteoblasts interact more dynamically with the underlying substrate, likely through the deposition of a specialized, OPN-rich transitional matrix that aids cell crawling.

A particularly interesting puzzle in our data was the mismatch at day 7 between phenotypic success and gene expression. On one hand, a 7-day exposure to these honeys sparked a massive surge in ALP enzymatic activity and rapid mineral nodule deposition, easily outperforming the standard chemical osteoinductive medium.

On the other hand, transcriptional analyses at this exact checkpoint showed a highly nuanced profile: while OPG and OPN spiked, the master transcription factor Runx2 remained steady, and osteocalcin (OCN) was slightly downregulated. In bone biology, this specific shift should not be misinterpreted as an inhibitory signal. OCN is a strict signature transcript of late-stage, post-mitotic mature osteoblasts [16]. Its temporary downtime, paired with stable Runx2 levels, indicates that within this early window, CH does not prematurely push cells into terminal exhaustion. Instead, it seems to stabilize a highly active, early-to-mid osteoprogenitor state tailored for rapid migration and initial matrix organization.

The marked induction of cathepsin K (cathK) introduces an apparent biochemical paradox, considering its traditional classification as a hallmark marker of osteoclastic bone resorption [17]. However, the recent literature [18,19,20] confirms that osteoblast lineages express baseline levels of cathK to manage initial extracellular matrix processing and collagen turnover. What guarantees the safety of our model is that the upstream regulatory components of the RANK/RANKL cascade remained completely unaffected by CH treatment. Because the RANKL signaling axis is the primary driver of osteoclast recruitment, its absolute stability—combined with the significant upregulation of the soluble decoy receptor osteoprotegerin (OPG)—strongly indicates that CH shifts the local RANKL/OPG balance toward bone protection, thereby limiting the activation of osteoclastogenic or bone-resorbing pathways.

The true turning point in our mechanistic breakdown is how coriander honey remodels intracellular calcium [Ca^2+^]_i_ dynamics. Calcium is a universal second messenger governing everything from cytoskeletal rearrangement to gene transcription [21]. Our confocal time-lapse imaging showed that CH triggers a rapid, sharp, and transient spike in [Ca^2+^]_i_. Through systematic pharmacological blocking experiments, we mapped the underlying circuitry of this signal. Our data suggest that the CH-evoked calcium surge involves a coordinated dual mechanism: it appears to rely on both an influx of extracellular calcium through membrane channels and an IP3-mediated internal release from the endoplasmic reticulum stores via PLC activation. Our mechanistic unraveling highlights the PLC-IP3/Ca^2+^ signaling cascade as a pivotal biochemical pathway that translates CH’s unique phytochemical stimulus into a coordinated functional output.

Naturally, these in vitro snapshots using hFOB 1.19 cells come with boundaries. An in vitro plate cannot replicate the systemic crosstalk or the complex vascular environment of a living bone. Factors like bioavailability, digestive breakdown, and the local concentration of honey bioactives reaching the bone site still need thorough validation.

Nonetheless, by proving that coriander honey directly orchestrates human osteoblast behavior through a clear PLC-IP3/Ca^2+^ pathway and a pro-anabolic gene shift, this study establishes a firm scientific foundation. These insights provide a compelling rationale for future in vivo studies to explore how coriander honey might be translated into practical, natural bioactive strategies for bone tissue engineering and regenerative medicine.

Although this study provides robust biochemical insights into the osteopromotive properties of coriander honey, certain limitations must be acknowledged. First, our mechanistic investigations rely on pharmacological inhibition strategies rather than genetic knockdown or knockout models; therefore, while the evidence strongly points toward the central involvement of the PLC-IP3 pathway, absolute causal relationships should be interpreted with caution. Second, this screening was restricted to the hFOB 1.19 cell line, a well-established and highly responsive model for committed human osteoblasts, but further studies on primary human mesenchymal stem cells (MSCs) will be required to assess the effects of CH on de novo osteoinduction. Finally, as an entirely in vitro study, these findings lack validation regarding the complex in vivo microenvironment, including pharmacokinetic parameters, bioavailability of honey flavonoids after digestion, and systemic bone remodeling dynamics. Future preclinical in vivo evaluations are strictly warranted to transition these promising cellular observations into translational bone repair applications.

## 4. Materials and Methods

### 4.1. Honeys

Three monofloral honey types—acacia (*Robinia pseudoacacia*), chestnut (*Castanea sativa*), and coriander (*Coriandrum sativum*)—were obtained from the beekeeping company “Davide e le sue api” located in Quargnento (Alessandria, Italy). All honeys were harvested within the Piedmont territory.

### 4.2. Folin–Ciocalteu Assay

To ascertain the overall polyphenol content in the honey samples examined, we employed the colorimetric in vitro Folin–Ciocalteu method. 100 μL of honey solution (1 gr in 3 mL of H_2_O) was added to 250 uL of the Folin–Ciocalteu (10% *v*/*v*). After 5 min, 100 uL of 7.5% (*w*/*v*) Na_2_CO_3_ was added and incubated for 15 min h at 50 °C. Samples were then rinsed with 2.5 mL of H_2_O [7].

The absorbance was measured at 620 nm in a plate reader (Infinite 200 Pro, Tecan, Wien, Austria). Standard curve was defined by known concentrations of gallic acid, and results were expressed in milligrams of gallic acid equivalents (mgGAE∙100 g^−1^).

### 4.3. Total Flavonoid Content

The total flavonoid content (TFC) of honey samples was assessed according to classic protocol. 1 mL of 0.5 g/mL honey solution and 1 mL of 2% aluminum chloride (AlCl_3_) were combined [7]. An aluminum-flavonoid combination was produced after 10 min at 25 °C of incubation. Using a plate reader (Infinite 200 Pro, Tecan), the complex’s creation was measured at 415 nm. Quercetin (0–200 µg/mL) served as the standard chemical in the creation of the calibration curve. The TFC was quantified as milligrams of quercetin equivalent in 0.5 g of honey and given as the mean value of triplicate experiments.

### 4.4. Free Radical Scavenging Activity

The antioxidant capacity of honeys was evaluated by the free radical scavenging ability of 2,2-Diphenyl-1-picrylhydrazyl (DPPH). The assay was performed by adding 1 mM DPPH solution to honeys (1 gr in 3 mL of H_2_O) followed by a 10 min incubation. The absorbance was determined at 512 nm in a plate reader (Infinite200 Pro, Tecan). The radical scavenging activity was compared to water [22].

### 4.5. Cell Culture

hFOB 1.19 (ATCC CRL-3602™) represents a homogeneous and fast-growing osteoblast cell line. It originated from human limb tissue obtained from a spontaneous miscarriage, which was then transfected with the temperature-sensitive expression vector pUCSVtsA58 and the neomycin resistance vector pSV2-neo [23,24,25]. Subsequent selection with 0.6 mg/mL G418 yielded stable clones. This cell line serves as a valuable model for investigating the intricate mechanisms of normal human osteoblast differentiation, exploring osteoblast physiology, and analyzing the impact of various signaling molecules, including hormones, growth factors, and cytokines, on osteoblast function and development.

For maintaining hFOB 1.19 cells, we used DMEM/Ham’s F-12 medium, enriched with 10% FBS, 2.5 mM of L-glutamine, and the selective agent G418 (0.3 mg/mL). We kept the cells in an environment of 34 °C and 5% CO_2_. Regular passaging was performed with a trypsin-EDTA solution as cells approached sub-confluence.

### 4.6. Scratch Wound Healing Assay

The migratory capacity of hFOB was assessed using a modified scratch wound healing assay [26]. Cells were seeded in 12-well tissue culture plates and allowed to reach ~90% confluence.

A sterile 200 μL pipette tip was then used to create a single linear scratch wound across the center of each well. Detached cells were removed by gently washing the wells twice with PBS. Subsequently, experimental treatments (e.g., various honey concentrations diluted) were added to the respective wells. Control wells received only complete medium.

Immediately after scratching (0 h) and at subsequent 24 h, images of the scratch wound were captured using an inverted phase-contrast microscope equipped with a digital camera. Care was taken to capture images at the exact same predefined locations for each well to ensure accurate comparison. The width of the scratch wound was measured using image analysis software (ImageJ 1.54g, NIH). Wound closure was quantified as the percentage of the initial wound area that had been covered by migrating cells. Experiments were performed in triplicate and repeated independently at least three times.

### 4.7. Alkaline Phosphatase (ALP) Assay

To assess ALP activity, we followed a previously established protocol [27]. Cells were cultured in 96-well plates and exposed to the various honeys, which were placed directly at the bottom of each well, for a duration of 7 days. At the conclusion of the experimental period, cells were incubated with a p-nitrophenol phosphate substrate for 10 min. The enzymatic reaction was then quenched using a 0.5 N NaOH solution. Finally, the concentration of the resulting p-nitrophenol was quantified spectrophotometrically at 405 nm using a microplate reader (Infinite Pro-200, Tecan).

### 4.8. Alizarin Red (AR) Assay

Cells were plated into 24-well plates and exposed to experimental conditions. Subsequent processing involved two PBS washes, followed by fixation with 10% paraformaldehyde for 15 min at room temperature. Mineralized matrix was visualized by incubating cells with AR for 20 min at room temperature, concluding with three washes with distilled water. For spectrophotometric quantification of the AR stain, cells were treated with 10% acetic acid for 30 min at room temperature. Cellular detachment was achieved mechanically using a cell scraper. The detached samples were then heated in microtubes at 85 °C for 10 min, and absorbance was measured at 405 nm on an Infinite 200 Pro plate reader (Tecan) [27].

### 4.9. Free Cytosolic Calcium Concentration ([Ca^2+^]_i_) Measurements

To measure free cytosolic Ca^2+^, hFOB cells were cultivated on glass-base dishes (Iwaki Glass, Inc., Tokyo, Japan). After achieving adequate attachment, cells were loaded in the dark for 30 min at 37 °C with 20 μM Fluo-3/AM, a cell-permeant fluorescent calcium indicator, utilizing a previously described loading buffer. After probe loading and washing steps, confocal time-lapse imaging was carried out. Excitation was performed with a 488 nm Ar laser (0.5% power to mitigate photobleaching), and emitted light was collected via a 505–550 nm bandpass filter.

A Zeiss LSM 510 confocal system, interfaced with a Zeiss Axiovert 100 M microscope (Carl Zeiss Inc., Oberkochen, Germany), was employed for data acquisition. Fluorescence from multiple cells was captured using a 20× objective, and mean ROI fluorescence was analyzed [28].

Fluo-3/AM calibration and subsequent [Ca^2+^] calculation were based on the Grynkiewicz equation [29]:

[Ca^2+^] = Kd(Fmax − F)(F − Fmin)

where Kd = 400 nmol/L. Fmin and Fmax values, representing the minimum and maximum fluorescence signals respectively, were established by sequentially treating cells with 500 μM A23187 for approximately 10 min, followed by 20 mM EDTA for 2 min.

### 4.10. Quantitative Reverse Transcriptase PCR (qRT-PCR)

For gene expression analysis, we extracted total RNA from the cells using the NucleoSpin RNAII Kit (Macherey-Nagel, Düren, Germany). cDNA was then synthesized from this RNA with the Transcriptor First Strand cDNA Synthesis Kit (Roche Diagnostics GmbH, Penzberg, Germany). qRT-PCR amplification took place on a CFX384 Real-Time PCR Detection System (Bio-Rad Laboratories, Hercules, CA, USA), employing Power Sybr Green Mastermix (Bio-Rad Lab) and KiCqStart^®^ SYBR^®^ Green Primers (primer details are in Table 1). Gene expression levels were quantified using the ΔΔCt method [30].

### 4.11. Statistical Analysis

Statistical analyses were performed using GraphPad Prism 8 (GraphPad Software Inc., San Diego, CA, USA). Depending on the experimental design and data distribution, statistical significance was determined by *t*-tests, one-way ANOVAs, or two-way ANOVAs. Appropriate post hoc corrections were applied as necessary. Comprehensive statistical details (e.g., sample size [n], specific test utilized, *p*-value, number of replicates) are provided in the respective Figure Legends.

### 4.12. Declaration of Generative AI and AI-Assisted Technologies in the Writing Process

The authors declare that generative AI and AI-assisted technologies were used during the preparation of this manuscript solely to improve the readability and language of the paper. After using this tool, the authors reviewed and edited the content as needed and took full responsibility for the accuracy and integrity of the final publication.

## 5. Conclusions

In summary, this comparative study establishes a direct, mechanistically grounded link between the floral source of honey and its unique capacity to orchestrate human osteoblast behavior. We demonstrate that honey’s intrinsic bioactivity is not generic; rather, its potency is heavily dictated by its botanical origin, which shapes a unique phytochemical and antioxidant footprint.

**Figure 7:**
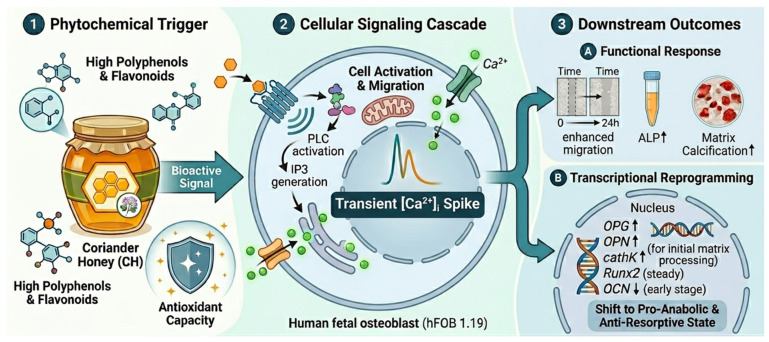
A proposed molecular mechanism of osteopromotive effects of coriander honey (CH) on human osteoblasts. The schematic summarizes the multiple effects of CH on hFOB 1.19 cells from the bioactive trigger to the downstream biological consequences in order. The upward-pointing arrows indicate an increase in the levels/expression of the corresponding protein/gene. The downward-pointing arrows indicate a decrease in the levels/expression of the corresponding protein/gene.

Among the Italian varieties screened, coriander honey (CH) emerged as exceptionally effective, showcasing a superior ability to accelerate osteoblast migration, early differentiation, and matrix calcification. Our mechanistic unraveling highlights the PLC-IP3/Ca^2+^ signaling cascade as a pivotal biochemical pathway that translates CH’s unique phytochemical stimulus into a coordinated functional output. By triggering this specific pathway and reshaping key pro-anabolic gene expression profiles, coriander honey successfully pushes human osteoblasts into a specialized, high-motility, and bone-protective state geared towards rapid matrix organization. These findings carry significant translational weight. By providing a solid molecular rationale for the use of coriander honey, this work justifies further in vivo and clinical exploration. We identify this specific floral variety as a highly promising, sustainable, and natural bioactive candidate for developing novel regenerative medicine strategies and tissue engineering scaffolds aimed at optimizing bone repair (Figure 7).

1. Phytochemical trigger: CH is rich in total phenolic and flavonoid contents that give it a strong antioxidant capacity. This phytochemical fingerprint is the main bioactive signal that protects osteoblasts against oxidative stress and triggers cellular responses.
2. Cellular signaling cascade: The bioactive compounds present in CH activate Phospholipase C (PLC) which produces inositol trisphosphate (IP3). IP3 binds to receptors on the endoplasmic reticulum (ER), causing transient release from intracellular calcium stores. This internal mobilization is associated with an influx of extracellular calcium through membrane channels giving rise to the characteristic transient spike in [Ca^2+^]_i_ that is the universal second messenger.
3. Downstream outcomes: The calcium signaling axis coordinates two parallel responses—(A) functional response, increased cell migration (scratch wound healing), increased Alkaline Phosphatase (ALP) enzymatic activity and increased Alizarin Red mineralization of the extracellular matrix. (B) Transcriptional reprogramming of specific genes: OPG, OPN and cathpK transcripts are significantly upregulated to promote matrix organization, cell motility and inhibition of resorption. Runx2 is steady and late-stage mature markers like OCN are temporarily quiescent during the early exposure window.

The diagram together shows how coriander honey is effective in inducing human osteoblasts into a state of high-motility, pro-anabolic progenitor, which is specialized in matrix organization and fast calcification.

This graphical synthesis of the mechanistic pathway was generated using the Gemini 1.5 Pro generative AI model (Google) based on the scientific results conceptualized by the authors.

## Figures and Tables

**Table 1:** List of primers used in qRT-PCR analysis.

cathK (gene CTSK)	5′-TGAGGCTTCTCTTGGTGTCCATAC-3′ 5′-AAAGGGTGTCATTACTGCGGG-3′
OCN (gene BGLAP)	5′-ACACTCCTCGCCCTATTG-3′ 5′-GATGTGGTCAGCCAACTC-3′
OPG (TNFRSF11B)	5′-GCTAACCTCACCTTCGAG-3′ 5′-TGATTGGACCTGGGTTACC-3′
OPN (gene SPP1)	5′-ACCATGAGAATTGCAGTGATTTGC-3′ 5′-ACCAGTTCATCAGATTC-3′
RANK (gene TNFRSF11A)	5′-TTAAGCCAGTGCTTCACGGG-3′ 5′-ACGTAGACCACGATGATGTCGC-3′
RANK-L (gene TNFSF11)	5′-ACGCAGATTTGCAGGACTCGAC-3′ 5′-TTCGTGCTCCCTCCTTTCATC-3′
RUNX2 (gene RUNX2)	5′-TGGTTACTGTCATGGCGGGTA-3′ 5′-TCTCAGATCGTTGAACCTTGCTA-3′
b-actin (gene ACTB)	5′-TCCCTGGAGAAGAGCTACGA-3′ 5′-AGCACTGTGTTGGCGTACAG-3′

## Data Availability

The original contributions presented in the study are included in the article, further inquiries can be directed to the corresponding author.

## Abbreviations

The following abbreviations are used in this manuscript:

ALP	Alike Phosphatase/Alkaline Phosphatase
AlCl_3_	Aluminum chloride
ANOVA	Analysis of variance
AR	Alizarin Red
ATCC	American Type Culture Collection
[Ca^2+^]_i_	Intracellular calcium concentration
cathK	Cathepsin K
CH	Coriander honey
DMEM/Ham’s F-12	Dulbecco’s Modified Eagle Medium/Nutrient Mixture F-12
DPPH	2,2-Diphenyl-1-picrylhydrazyl assay
EDTA	Ethylenediaminetetraacetic acid
ER	Endoplasmic reticulum
FBS	Fetal bovine serum
hFOB/hOBs	Human fetal osteoblastic cells/Human osteoblasts
IP3	Inositol trisphosphate
IP3R	Inositol trisphosphate receptor
Kd	Dissociation constant
MGO	Methylglyoxal
mgGAE	Milligrams of gallic acid equivalents
mRNA	Messenger ribonucleic acid
Na_2_CO_3_	Sodium carbonate
NaOH	Sodium hydroxide
OCN	Osteocalcin
OI	Osteoinductive medium
OPG	Osteoprotegerin
OPN	Osteopontin
PBS	Phosphate-buffered saline
PLC	Phospholipase C
qRT-PCR	Quantitative reverse transcriptase polymerase chain reaction
RANK	Receptor activator of nuclear factor κB
RANKL	Receptor activator of nuclear factor κB ligand
ROI	Region of interest
ROS	Reactive oxygen species
Runx2	Runt-related transcription factor 2
SD	Standard deviation
SEM	Standard error of the mean
TFC	Total flavonoid content
U73122	1-(6-((17β-3-Methoxyestra-1,3,5(10)-trien-17-yl)amino)hexyl)-1H-pyrrole-2,5-dione (PLC inhibitor)
*w*/*v*	Weight per volume
*v*/*v*	Volume per volume

## References

[1] Florencio-Silva R., Sasso G.R., Sasso-Cerri E., Simões M.J., Cerri P.S. (2015). Biology of Bone Tissue: Structure, Function, and Factors That Influence Bone Cells. BioMed Res. Int..

[2] Ilyas S., Lee J., Lee D. (2024). Emerging Roles of Natural Compounds in Osteoporosis: Regulation, Molecular Mechanisms and Bone Regeneration. Pharmaceuticals.

[3] Qu Z., Zhao S., Zhang Y., Wang X., Yan L. (2024). Natural Compounds for Bone Remodeling: Targeting osteoblasts and relevant signaling pathways. Biomed. Pharmacother..

[4] Bonsignore G., Martinotti S., Ranzato E. (2024). Honey Bioactive Molecules: There Is a World Beyond the Sugars. BioTech.

[5] Rossi M., Marrazzo P. (2021). The Potential of Honeybee Products for Biomaterial Applications. Biomimetics.

[6] Alvarez-Suarez J.M., Gasparrini M., Forbes-Hernández T.Y., Mazzoni L., Giampieri F. (2014). The Composition and Biological Activity of Honey: A Focus on Manuka Honey. Foods.

[7] Martinotti S., Bonsignore G., Patrone M., Ranzato E. (2025). Correlation between Honey Parameters and Wound Healing Properties: The Case of Piedmont (Italy) Samples. Curr. Pharm. Biotechnol..

[8] Semicheva A., Ersoy U., Vasilaki A., Myrtziou I., Kanakis I. (2024). Defining the Most Potent Osteoinductive Culture Conditions for MC3T3-E1 Cells Reveals No Implication of Oxidative Stress or Energy Metabolism. Int. J. Mol. Sci..

[9] Singh V. (2017). Medicinal plants and bone healing. Natl. J. Maxillofac. Surg..

[10] Kamaruzzaman M.A., Chin K.Y., Mohd Ramli E.S. (2019). A Review of Potential Beneficial Effects of Honey on Bone Health. Evid. Based Complement. Altern. Med..

[11] Kamboj R., Sandhu R.S., Kaler R.S.S., Bera M.B., Nanda V. (2019). Optimization of process parameters on hydroxymethylfurfural content, diastase and invertase activity of coriander honey. J. Food Sci. Technol..

[12] Domazetovic V., Marcucci G., Iantomasi T., Brandi M.L., Vincenzini M.T. (2017). Oxidative stress in bone remodeling: Role of antioxidants. Clin. Cases Miner. Bone Metab..

[13] Cervellati C., Bonaccorsi G., Cremonini E., Romani A., Fila E., Castaldini M.C., Ferrazzini S., Giganti M., Massari L. (2014). Oxidative stress and bone resorption interplay as a possible trigger for postmenopausal osteoporosis. BioMed Res. Int..

[14] Sharifi M.A., Yasin N.F., Kamarul T., Sharifi A.M. (2025). The regenerative journey: Exploring stem cell roles from injury detection to tissue repair. Stem Cell Res. Ther..

[15] Karasalih B., Duman H., Bechelany M., Karav S. (2025). Osteopontin: Its Properties, Recent Studies, and Potential Applications. Int. J. Mol. Sci..

[16] da Silva Sasso G.R., Florencio-Silva R., de Pizzol-Júnior J.P., Gil C.D., Simões M.J., Sasso-Cerri E., Cerri P.S. (2023). Additional Insights Into the Role of Osteocalcin in Osteoblast Differentiation and in the Early Steps of Developing Alveolar Process of Rat Molars. J. Histochem. Cytochem..

[17] Panwar P., Olesen J.B., Delaisse J.M., Søe K., Brömme D. (2025). Cathepsin K inhibitors promote osteoclast-osteoblast communication and engagement of osteogenesis. JBMR Plus.

[18] Kalajzic I., Staal A., Yang W.P., Wu Y., Johnson S.E., Feyen J.H., Krueger W., Maye P., Yu F., Zhao Y. (2005). Expression profile of osteoblast lineage at defined stages of differentiation. J. Biol. Chem..

[19] Morko J., Kiviranta R., Mulari M.T., Ivaska K.K., Väänänen H.K., Vuorio E., Laitala-Leinonen T. (2009). Overexpression of cathepsin K accelerates the resorption cycle and osteoblast differentiation in vitro. Bone.

[20] Calabrese G., Bennett B.J., Orozco L., Kang H.M., Eskin E., Dombret C., De Backer O., Lusis A.J., Farber C.R. (2012). Systems genetic analysis of osteoblast-lineage cells. PLoS Genet..

[21] Carafoli E., Krebs J. (2016). Why Calcium? How Calcium Became the Best Communicator. J. Biol. Chem..

[22] Chua L.S., Rahaman N.L., Adnan N.A., Eddie Tan T.T. (2013). Antioxidant Activity of Three Honey Samples in relation with Their Biochemical Components. J. Anal. Methods Chem..

[23] Jablonská E., Mrázková L., Kubásek J., Vojtěch D., Paulin I., Ruml T., Lipov J. (2024). Characterization of hFOB 1.19 Cell Line for Studying Zn-Based Degradable Metallic Biomaterials. Materials.

[24] Harris S.A., Enger R.J., Riggs B.L., Spelsberg T.C. (1995). Development and characterization of a conditionally immortalized human fetal osteoblastic cell line. J. Bone Miner. Res..

[25] Bousch J.F., Beyersdorf C., Schultz K., Schnitker M., Suschek C.V., Maus U. (2025). Comparison of Primary Human Osteoblast-like Cells and hFOB 1.19 Cells: Contrasting Effects of Proinflammatory Cytokines. Cells.

[26] Martinotti S., Ranzato E. (2020). Scratch Wound Healing Assay. Methods Mol. Biol..

[27] Antenucci S., Panzella L., Farina H., Ortenzi M.A., Caneva E., Martinotti S., Ranzato E., Burlando B., d’Ischia M., Napolitano A. (2016). Powering tyrosol antioxidant capacity and osteogenic activity by biocatalytic polymerization. RSC Adv..

[28] Ranzato E., Bonsignore G., Patrone M., Martinotti S. (2021). Endothelial and Vascular Health: A Tale of Honey, H. Cells.

[29] Grynkiewicz G., Poenie M., Tsien R.Y. (1985). A new generation of Ca2+ indicators with greatly improved fluorescence properties. J. Biol. Chem..

[30] Ranzato E., Bonsignore G., Martinotti S. (2022). ER Stress Response and Induction of Apoptosis in Malignant Pleural Mesothelioma: The Achilles Heel Targeted by the Anticancer Ruthenium Drug BOLD-100. Cancers.

